# Specific MAPK-Associated MicroRNAs in Serum Differentiate Pancreatic Cancer from Autoimmune Pancreatitis

**DOI:** 10.1371/journal.pone.0158669

**Published:** 2016-07-05

**Authors:** Manabu Akamatsu, Naohiko Makino, Yushi Ikeda, Akiko Matsuda, Miho Ito, Yasuharu Kakizaki, Yoshihiko Saito, Tetsuya Ishizawa, Toshikazu Kobayashi, Toru Furukawa, Yoshiyuki Ueno

**Affiliations:** 1 Department of Gastroenterology, Faculty of Medicine, Yamagata University, Yamagata, Japan; 2 Institute for Integrated Medical Sciences, Tokyo Women’s Medical University, Tokyo, Japan; University of Pittsburgh, UNITED STATES

## Abstract

Pancreatic ductal adenocarcinoma (PDAC) is difficult to distinguish from autoimmune pancreatitis (AIP) because of their clinical and radiological similarities, and therefore simple and minimally invasive surrogate markers for differential diagnosis would be useful. In our previous studies, we identified four microRNAs (miRNAs)–miR-7, miR-34a, miR-181d, and miR-193b –as MAPK-associated microRNAs whose expression was altered significantly with upregulation of the MAPK signaling pathway. Recently it has been reported that these miRNAs could be used as biomarkers in serum samples for accurate diagnosis of pancreatic lesions. The aim of the present study was to evaluate whether these MAPK-associated miRNAs in serum could be used as biomarkers for differentiating PDAC from AIP. We enrolled 69 patients with PDAC, 26 with intraductal papillary mucinous neoplasm (IPMN) and 15 with AIP. The expression of MAPK-associated miRNAs in serum was measured by quantitative real-time PCR. The 2^-ΔCT^ method was used to quantify the expression of miRNAs, and the data were normalized using spiked-in synthetic cel-miR-39. Patients with PDAC or IPMN showed significantly higher amounts of serum MAPK-associated miRNAs than those with AIP (p<0.009 for miR-7, p<0.002 for miR-34a, p<0.001 for miR-181d, p<0.002 for miR-193b). ROC curve analysis demonstrated that these miRNAs had an area under the ROC curve (AUC) of 0.723–0.882 for differentiation between PDAC or IPMN from AIP. Furthermore, serum miR-181d was significantly associated with the presence of metastasis in patients with PDA (p = 0.014). Serum MAPK-associated miRNAs could be novel noninvasive biomarkers for differentiation between PDAC or IPMN and AIP.

## Introduction

Pancreatic cancer is one of the major causes of cancer death worldwide [1, 2]. Despite advances in diagnostic and therapeutic techniques, the 5-year survival rate of patients with pancreatic cancer remains less than 10% [3]. Autoimmune pancreatitis (AIP) is a rare form of chronic pancreatitis for which both the prognosis and treatment differ markedly from those for pancreatic cancer. However, pancreatic cancer is sometimes difficult to distinguish from AIP because of their clinical and radiological similarities [4], and therefore accurate diagnosis is essential. Although the development of endoscopic ultrasonography-guided fine-needle aspiration (EUS-FNA) has made it possible to obtain samples of pancreatic tissue in diagnostic procedures, the technique has certain limitations, such as invasiveness and difficulty in obtaining a sufficient number of cells, and therefore more effective and minimally invasive diagnostic measures would be useful.

Pancreatic ductal adenocarcinoma (PDAC), a common type of pancreatic cancer, is characterized by frequent gain-of-function mutations in the KRAS gene, which encodes a GDP/GTP-binding protein that transmits stimulatory signals to several downstream cascades, including the mitogen-activated protein kinase (MAPK) pathways [5, 6]. In PDAC, MAPK is constitutively activated by mutation of KRAS and synergistic epigenetic inactivation of DUSP6 [7].

MicroRNAs (miRNAs) are endogenous small non-coding RNAs that regulate the expression of target genes by interfering with transcription and/or translation [8, 9]. MiRNAs play important roles in various diseases including cancers [10, 11], and their application for diagnosis or treatment is anticipated. A number of reports have indicated that aberrant expression of miRNAs may contribute to development and progression of PDAC [11, 12]. MiRNAs are stably present in exosomes in serum, and their utility for early diagnosis of PDAC has been reported [13]. However, little is known about the utility of miRNAs as diagnostic biomarkers for distinguishing between PDAC and AIP.

In our previous study, we identified four miRNAs–miR-7, miR-34a, miR-181d and miR-193b –that are specifically associated with constitutive activation of MAPK in pancreatic cancer cells [14]. We demonstrated that these miRNAs, most notably miR-193b, were able to suppress the proliferation of pancreatic cancer cells, and using a reporter assay we confirmed that miR-193b inhibited the translation of multiple genes associated with cancer phenotypes. We termed these miRNAs MAPK-associated miRNAs. Because the essential difference between PDAC and AIP may depend on whether or not MAPK is preferentially activated, we hypothesized that these MAPK-associated miRNAs might be useful as serum biomarkers for differentiating PDAC from AIP.

The aim of the present study was to assess the diagnostic value of MAPK-associated miRNAs in serum for differentiating PDAC from AIP. To evaluate whether these miRNAs also show changes in expression depending on activation of the RAS/MAPK pathway *in vivo*, we compared their levels of expression in three groups of patients with pancreatic disease–PDAC, intraductal papillary mucinous neoplasm (IPMN) and AIP–showing various frequencies of KRAS gene mutation. Around 90–95% of cases of PDAC harbor KRAS gene mutation [5, 15], whereas such mutation is present in 50% of cases of IPMN [16] and absent in AIP [17, 18].

## Materials and Methods

### Patients

For this study we enrolled 69 patients with PDAC, 26 patients with IPMN, and 15 patients with AIP diagnosed at Yamagata University Faculty of Medicine between January 1, 2007 and December 31, 2012. All serum samples were obtained before the start of treatment. Diagnosis was based on pathologic examination of surgical specimens, whereas for unresectable cases it was based on comprehensive assessment of the clinical course, imaging examinations, and EUS-FNA. All IPMN cases were resected and all were adenomas. The average ages of the patients in the PDAC, IPMN and AIP groups were 68.2 (51–86) years, 67.2 (47–79) years, and 68.7 (52–87) years, and the male to female ratios were 45:24, 19:7, and 12:3, respectively. There were no significant differences in the ages and male to female ratios among the three groups (p = 0.844, ANOVA; p = 0.441, Pearson’s chi-squared test). Clinical data of the patients are presented in Table 1.

**Table 1:** Patient information.

Characteristics	AIP (n = 15)	IPMN (n = 26)	PDAC (n = 69)	
**Gender**				P value
Male	12	19	45	0.441^a^
Female	3	7	24	
**Age at diagnosis**				
Mean±SD	68.7±11	67.2±7	68.2±8	0.844^b^
Median (range)	69 (52–87)	68 (47–79)	68 (51–86)	
**TNM stage**				
I			3	
II			0	
III			14	
IVa			17	
IVb			35	
**Tumor location**				
head (including uncus)		11	34	
body		11	26	
tail		4	8	
unknown			1	
**Subtype**				
diffuse	3			
segmental/focal	12			
**Operation**				
resectable		26	24	
unresectable		0	45	

AIP, autoimmune pancreatitis; IPMN, intraductal papillary mucinous neoplasm; PDAC, pancreatic ductal adenocarcinoma; SD, standard deviation.

^a^ ANOVA,

^b^ Pearson’s chi-squared test.

### RNA isolation

Venous blood samples were collected from each patient and processed within 1 hour. Separation of the serum was accomplished by centrifugation at 3000 rpm for 10 min at room temperature. The serum supernatant was recovered and stored at -80°C until analysis.

Total RNA (which includes small RNAs) was isolated from 200 μL of serum using TRIzol LS Reagent (Ambion, Austin, TX, USA) following the manufacturer’s protocol. To normalize for technical variation in the RNA extraction step, 10 μL of synthetic cel-miR-39 (0.5 fmol/μL) was added routinely to each sample after initial denaturation of the serum [19–21].

### Reverse transcription

Reverse transcription was conducted using a Taqman MicroRNA RT Kit (Applied Biosystems, Foster City, CA, USA) and a miRNA-specific stem-loop primer (Applied Biosystems). The reverse transcriptase reaction mixture (15 μL) contained 5 μL RNA sample, 3 μL RT-primer, 1.5 μL 10x Reverse Transcription Buffer, 1 μL Multiscribe^™^ RT enzyme, 0.15 μL dNTP mix (with dTTP), 0.19 μL RNase inhibitor and 4.16 μL DEPC-treated water. The total reaction mixture was incubated in a GeneAmp PCR System 2700 (Applied Biosystems) for 30 min at 16°C, 30 min at 42°C, and 5 min at 85°C, and maintained at 4°C.

### Quantitative real-time PCR (qRT-PCR)

qRT-PCR was carried out in duplicate using Taqman miRNA assays (Applied Biosystems) on a 7500 Fast Real-Time PCR System (Applied Biosystems). The PCR mixture (10 μL) contained 5 μL Taqman Universal PCR Mastermix (Applied Biosystems), 3.83 μL nuclease-free DEPC-treated water, 0.5 μL Taqman miRNA assay (Applied Biosystems), and 0.67 μL reverse-transcribed products. The reaction conditions were 40 amplification cycles of 95°C for 15 s and 60°C for 1 min in a 96-well optical plate. Cycle threshold (Ct) values were calculated by using same threshold cut-off values for each assay to prevent plate-to-plate variations while analyzing the data with the 7500 software package v.2.0.6 (Applied Biosystems). The expression levels of miRNAs were normalized to spiked-in cel-miR-39, and were calculated utilizing the 2^-ΔCt^ method [22]. ΔCt was calculated by subtracting the Ct values of cel-miR-39 from the Ct values of the miRNA of interest.

### Statistical analysis

The Mann-Whitney test and Kruskal-Wallis analyses were used to evaluate statistical differences in serum miRNA levels between unpaired groups and multiple comparison groups, respectively. Receiver operating characteristic (ROC) curve analysis was performed to determine the diagnostic performance of each miRNA for differentiating patients with PDAC from those with AIP. Sensitivity against 100% minus specificity was plotted at each cutoff threshold, and the area under the curve (AUC) values reflecting the probability of correctly distinguishing PDAC from AIP was computed [22]. All P-values were two-sided and differences at P<0.05 were considered statistically significant. All statistical analyses were performed with StatMate v. 4.01 (ATMS Inc.).

### Ethics

The present study was performed in accordance with the Declaration of Helsinki, and approved by the research ethics board of Yamagata University Faculty of Medicine. All patients participating in this study provided written informed consent before sample collection.

## Results

**Fig 1:**
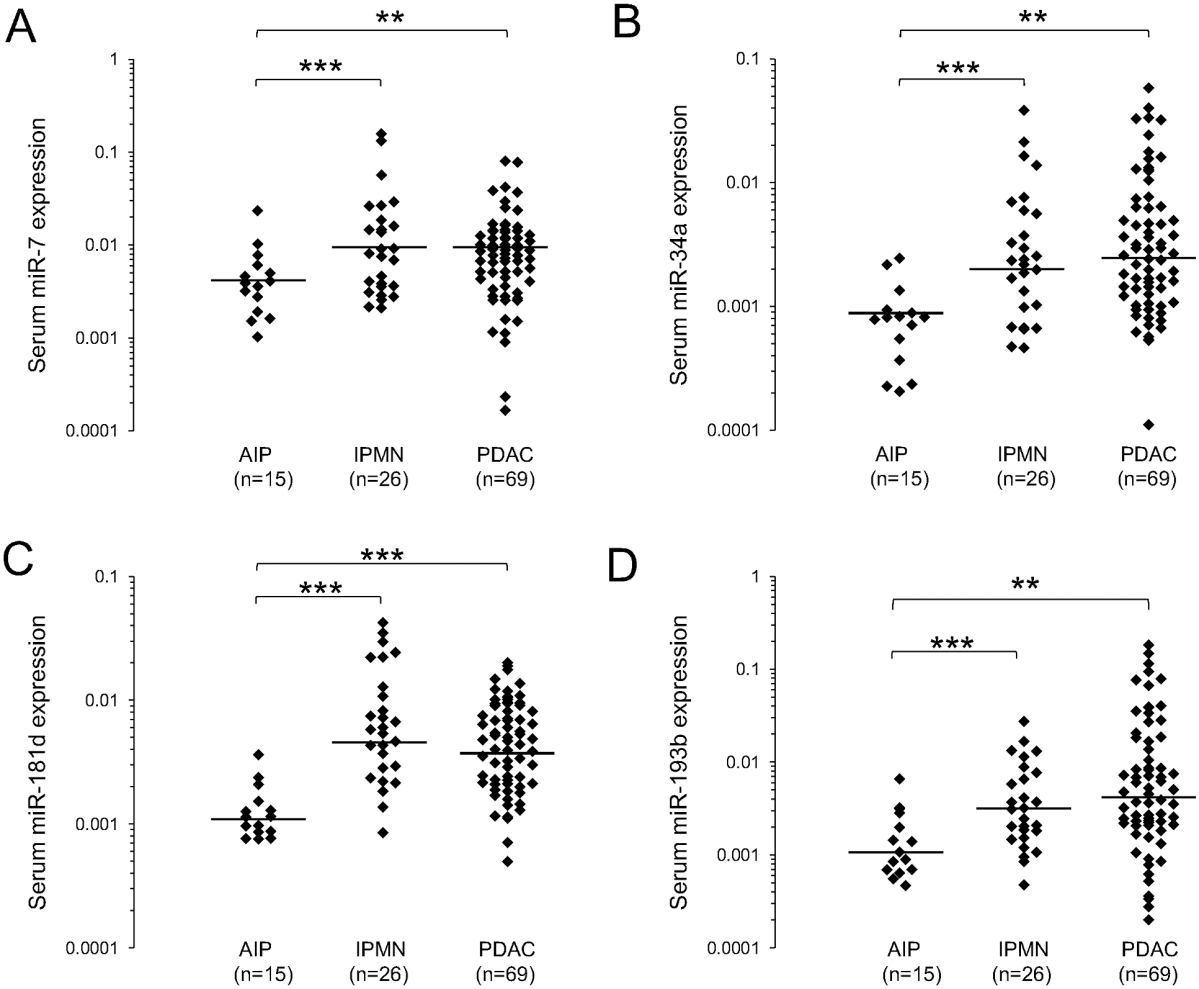
Serum expression of miRNAs in patients with pancreatic diseases. Scatter plots of serum miRNA expression in AIP (n = 15), IPMN (n = 26) and PDAC patients (n = 69). The Y-axis represents the relative expression of the miRNAs normalized to cel-miR-39. The line represents the median value. The significance of differences was determined using Mann-Whitney test (***P < .001: **P < .01). (A) miR-7 expression. (B) miR-34a expression. (C) miR-181d expression. (D) miR-193b expression. AIP, autoimmune pancreatitis; IPMN, intraductal papillary mucinous neoplasm; PDAC, pancreatic ductal adenocarcinoma.

In an initial analysis, we compared the expression levels of MAPK-associated miRNAs in serum among the three patient groups, i.e., those with PDAC, IPMN, and AIP (Fig 1). The PDAC and IPMN groups showed significantly higher amounts of all miRNAs than the AIP group. The fold change in the median value for miR-7 was 1.8 in the PDAC group (p = 0.009) and 2.4 in the IPMN group (p<0.001) relative to that in the AIP group. The corresponding value for miR-34a was 3.7 in the PDAC group (p<0.002) and 3.3 in the IPMN group (p<0.001). The corresponding values for miR-181d and miR-193b were 3.5 (p<0.001) and 5.1 (p<0.001), and 4.1 (p<0.002) and 2.5 (p<0.001), respectively. There were no significant differences in these values between PDAC and IPMN.

**Fig 2:**
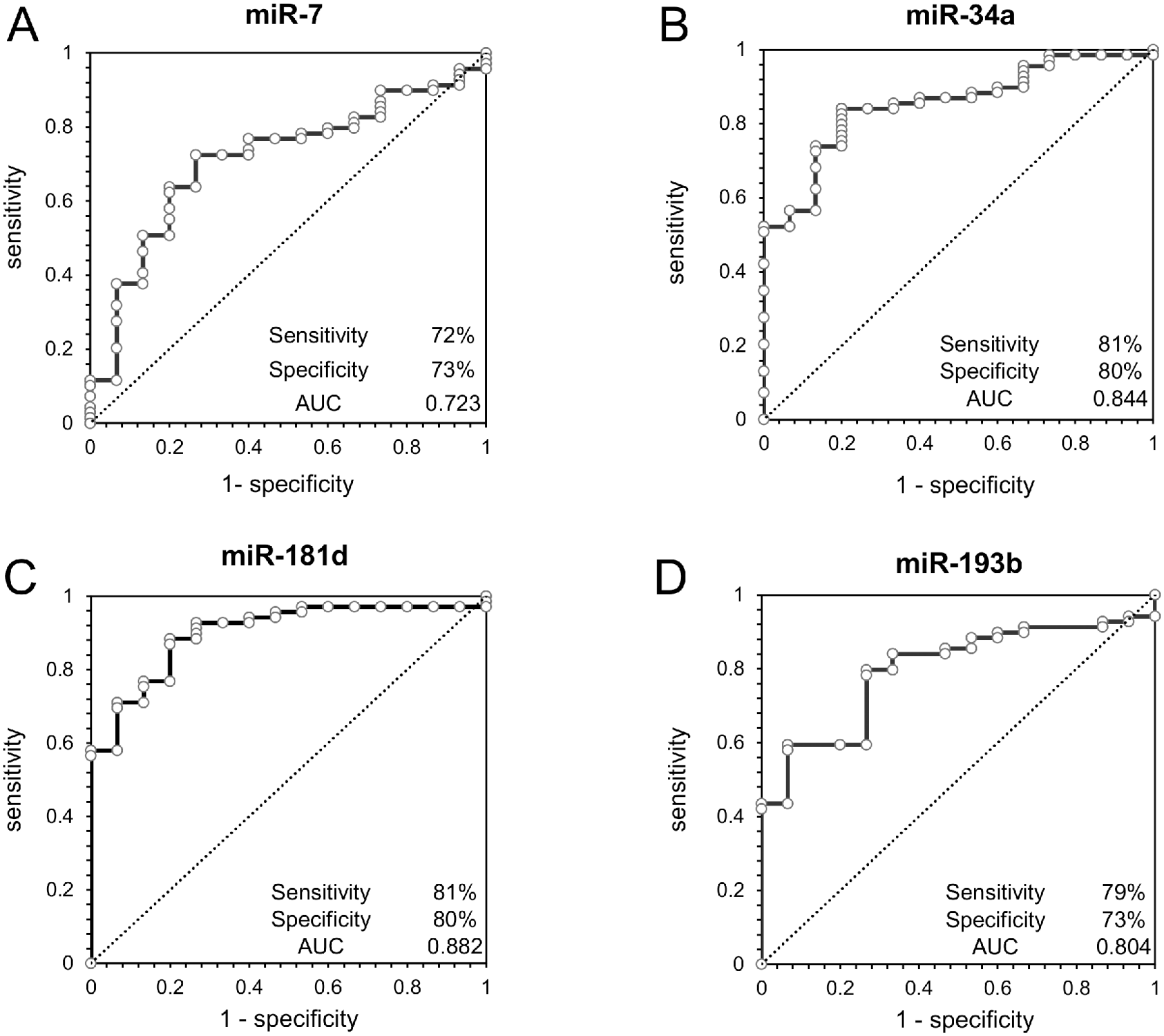
Receiver operating characteristic curve analysis using serum microRNAs ((A) miR-7, (B) miR-34a, (C) miR-181d, (D) miR-193b) for distinguishing PDAC from AIP.

Next, we performed ROC curve analysis of these MAPK-associated miRNAs in serum (Fig 2). Serum miR-7 yielded an AUC value of 0.723 (95% confidence interval [CI] = 0.603 to 0.844), with 72% sensitivity and 73% specificity for distinguishing PDAC from AIP. MiR-34a yielded an AUC value of 0.844 (95% CI = 0.754 to 0.935), with 81% sensitivity and 80% specificity. MiR-181d yielded an AUC value of 0.882 (95% CI = 0.813 to 0.952), with 81% sensitivity and 80% specificity. MiR-193b yielded an AUC value of 0.804 (95% CI = 0.718 to 0.892), with 79% sensitivity and 73% specificity.

**Fig 3:**
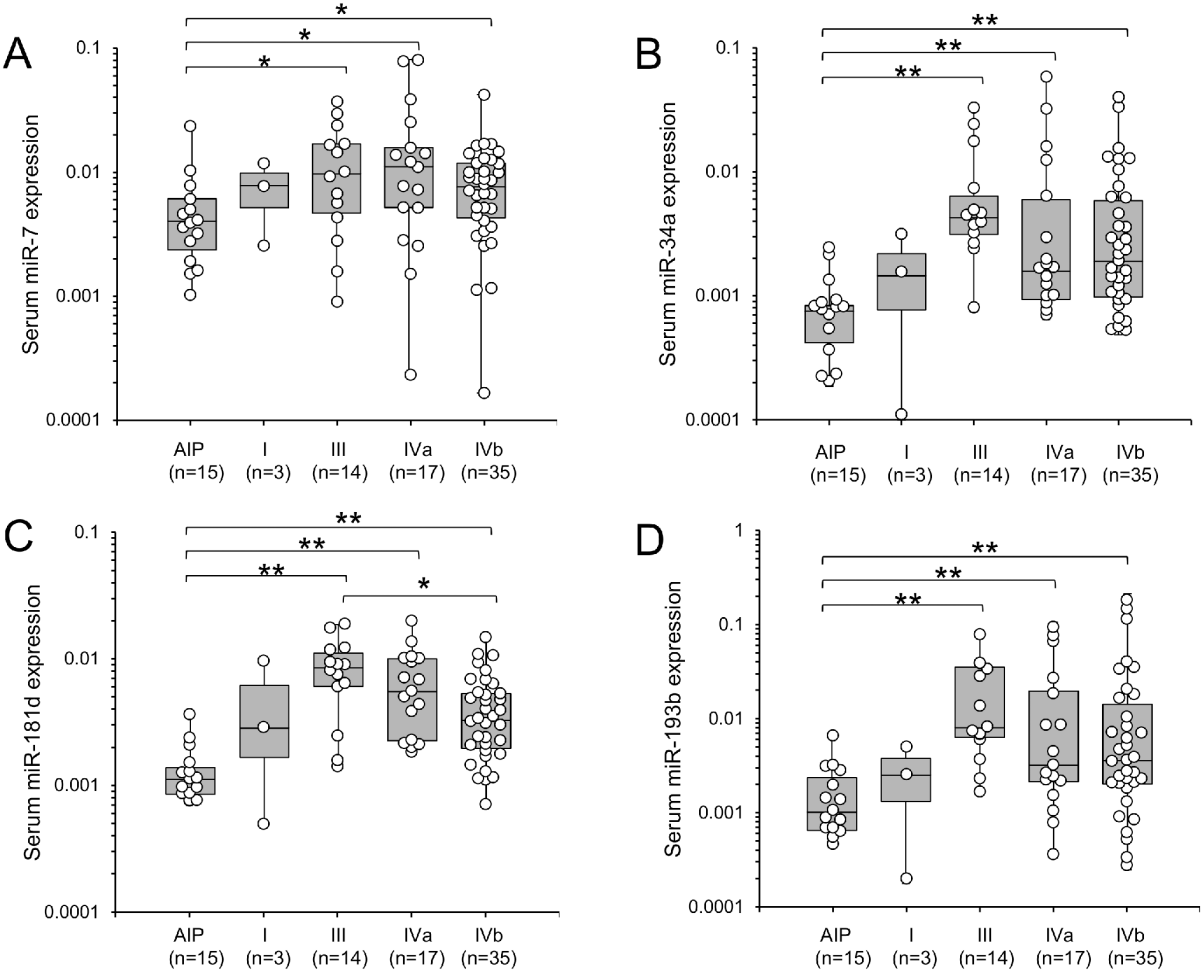
Serum miRNA expression in PDAC at different Tumor Node Metastasis (TNM) stages. The Y-axis (log_10_ scale) represents the relative expression of each miRNA normalized to cel-miR-39. Boxes represent the interquartile range, and the horizontal line across each box indicates the median value. The significance of differences was determined using Mann-Whitney test (**P < .01: *P < .05). (A) miR-7 expression. (B) miR-34a expression. (C) miR-181d expression. (D) miR-193b expression. PDAC, pancreatic ductal adenocarcinoma; AIP, autoimmune pancreatitis.

**Fig 4:**
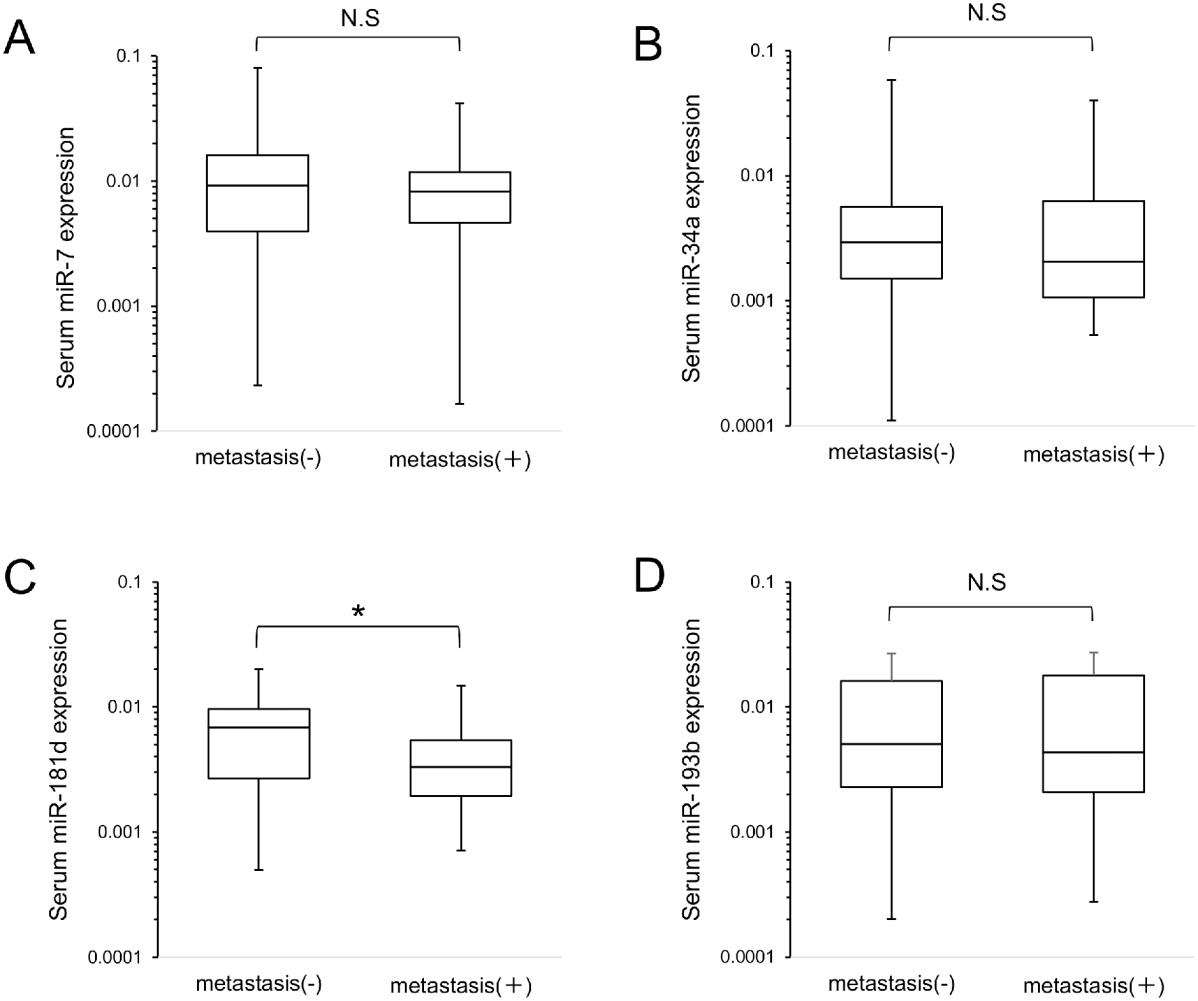
Serum miRNA expression in PDAC patients with distant metastasis (n = 35) and PDAC patients without distant metastasis (n = 34). The Y-axis (log_10_ scale) represents the relative expression of each miRNA normalized to cel-miR-39. Boxes represent the interquartile range, and the horizontal line across each box indicates the median value (A) miR-7 expression. (B) miR-34a expression. (C) miR-181d expression. (D) miR-193b expression. The significance of differences was determined using Mann-Whitney test (*P < .05). N.S, not statistically significant. PDAC, pancreatic ductal adenocarcinoma.

We then examined the relationship between these miRNAs and the clinical stage of PDAC, but no specific associations were evident for miR-7, miR-34a and miR-193b (Fig 3); only miR-181d showed a significant difference between stage III and IVb (p = 0.01). We then examined the association between these miRNAs and metastasis of PDAC (Fig 4). Metastasis was found in 35 patients, and no metastasis was observed in the remaining 34. We found that only miR-181d showed a significant association, the serum miR-181d level being significantly lower in patients with metastasis (p = 0.014).

## Discussion

PDAC is a malignant disease with a poor prognosis, whereas AIP is a benign inflammatory disease that mimics PDAC both clinically and radiologically. An elevated serum level of IgG4 is a characteristic feature of AIP, but mild elevation of serum IgG4 is also seen in 10% of PDAC cases and therefore cannot be used alone to distinguish AIP from PDAC [23, 24]. Response to steroids can help to confirm a suspected case of AIP, but care must be taken to avoid confusing this with spontaneous radiological improvement in pancreatic cancer-induced pancreatitis. In recent years, the use of EUS-FNA has become widespread, and various reports have indicated that it is useful for differentiation between the two diseases [25, 26]. However, definitive diagnosis of AIP is still difficult due to the small size of the specimens obtained by EUS-FNA [27, 28]. Attempts have been made to improve the rate of PDAC diagnosis by genetic screening using EUS-FNA specimens [17], but EUS-FNA itself may sometimes be difficult, depending on the part of the lesion sampled and the neighboring bloodstream. For these reasons, some other means of distinguishing PDAC from AIP using less invasive methods would be desirable. The present study demonstrated a significant difference in expression of serum miRNAs between PDAC and AIP.

To assess the diagnostic value of MAPK-associated miRNAs, we performed ROC curve analysis. All of the tested miRNAs showed good sensitivity and specificity for distinguishing PDAC from AIP, suggesting that they could potentially contribute to more precise diagnosis of PDAC. However, as the number of AIP cases investigated was small, further studies will be needed.

We have already reported that these four MAPK-associated miRNAs (miR-7, miR-34a, miR-181d and miR-193b) are characterized by a change in their expression levels depending on activation of the RAS/MAPK pathway, and act as tumor suppressors *in vitro* [13]. Other reports have indicated that these four miRNAs have a tumor-suppressor function, but only miR-34a is reportedly associated with PDAC [29, 30]. We clarified that these miRNAs are up-regulated in both PDAC and IPMN, perhaps reflecting the presence of KRAS gene mutation in those neoplasms. We were unable to specifically confirm KRAS gene mutation in surgical specimens from all of the patients. Therefore, we attempted to indirectly evaluate the association of changes in miRNA expression with KRAS gene mutation.

There was no convincing evidence for a relationship between the expression levels of three of the miRNAs (miR-7, miR-34a and miR-193b) and TNM stage in PDAC. The KRAS gene mutation rate is not correlated with the progression of PDAC because mutation is already present at the PanIN stage [31]. Therefore, this result was considered reasonable, and the observed abnormality of MAPK-associated miRNA expression in serum may have simply reflected the presence of KRAS gene mutation in the tumor. On the other hand, because a significant difference in the expression of miR-181d was confirmed between stages III and IVb, we hypothesized that miR-181d might play a role in metastasis. To examine this possibility, we divided the PDAC patients into two subgroups according to the presence or absence of distant metastasis, and checked the levels of miRNA expression in the two subgroups. Although the statistical analysis we employed may not have been wholly applicable for multiple comparisons, only miR-181d was found to be significantly down-regulated in the metastasis group, possibly indicating that miR-181d plays a role in PDAC metastasis. As miR-181d has not yet been investigated in the context of oncology, a detailed functional analysis of this miRNA would be informative. Although the role of miR-181d in metastasis is unclear, a previous report has indicated that it acts as a tumor suppressor in glioma by targeting KRAS [32]. This suggests that miR-181d may form a negative feedback loop to regulate the RAS/MAPK pathway in PDAC; in other words up-regulation of serum MAPK-associated miRNA expression may be a physiological reaction against pancreatic neoplasms. Thus, the lower levels of miR-181d that we observed among those with distant metastasis versus no distant metastasis are consistent with a loss of this negative feedback function.

In conclusion, we have determined the expression profiles of MAPK-associated miRNAs in serum from patients with various pancreatic diseases, and demonstrated a significant difference in miRNA expression between AIP–a non-neoplastic disease–and PDAC and IPMN, which are both neoplastic. Abnormality of MAPK-associated miRNAs in serum may reflect the presence of KRAS gene mutation, and therefore these miRNAs may have potential as novel noninvasive biomarkers for differentiating PDAC from AIP. Future studies are needed to clarify the role of these circulating miRNAs.
